# The Role of *Pectobacterium atrosepticum* Exopolysaccharides in Plant–Pathogen Interactions

**DOI:** 10.3390/ijms222312781

**Published:** 2021-11-26

**Authors:** Bakhtiyar Islamov, Olga Petrova, Polina Mikshina, Aidar Kadyirov, Vladimir Vorob’ev, Yuri Gogolev, Vladimir Gorshkov

**Affiliations:** 1 Kazan Institute of Biochemistry and Biophysics, FRC Kazan Scientific Center of RAS, 420111 Kazan, Russia; bah-islam80@mail.ru (B.I.); poe60@mail.ru (O.P.); p.mikshina@gmail.com (P.M.); vorobyev@kibb.knc.ru (V.V.); gogolev.yuri@gmail.com (Y.G.); 2 Laboratory of Plant Infectious Diseases, FRC Kazan Scientific Center of RAS, 420111 Kazan, Russia; 3 Institute of Power Engineering and Advanced Technologies, FRC Kazan Scientific Center of RAS, 420111 Kazan, Russia; aidarik@rambler.ru

**Keywords:** bacterial emboli, detoxification of reactive oxygen species, exopolysaccharides, immunosuppressors, molecular aggregates, *Pectobacterium*, viscosity

## Abstract

The phytopathogenic bacterium *Pectobacterium atrosepticum* (*Pba*), one of the members of the soft rot *Pectobacteriaceae*, forms biofilm-like structures known as bacterial emboli when colonizing the primary xylem vessels of the host plants. The initial extracellular matrix of the bacterial emboli is composed of the host plant’s pectic polysaccharides, which are gradually substituted by the *Pba*-produced exopolysaccharides (*Pba* EPS) as the bacterial emboli “mature”. No information about the properties of *Pba* EPS and their possible roles in *Pba*-plant interactions has so far been obtained. We have shown that *Pba* EPS possess physical properties that can promote the maintenance of the structural integrity of bacterial emboli. These polymers increase the viscosity of liquids and form large supramolecular aggregates. The formation of *Pba* EPS aggregates is provided (at least partly) by the acetyl groups of the *Pba* EPS molecules. Besides, *Pba* EPS scavenge reactive oxygen species (ROS), the accumulation of which is known to be associated with the formation of bacterial emboli. In addition, *Pba* EPS act as suppressors of the quantitative immunity of plants, repressing PAMP-induced reactions; this property is partly lost in the deacetylated form of *Pba* EPS. Overall, our study shows that *Pba* EPS play structural, protective, and immunosuppressive roles during *Pba*–plant interactions and thus should be considered as virulence factors of these bacteria.

## 1. Introduction

Plant diseases caused by the members of the soft rot *Pectobacteriaceae* (SRP) usually manifest as the destruction of the outer parenchymatous tissues of the host plant. However, these bacteria, in addition to parenchymatous tissues, extensively colonize xylem vessels [1,2,3,4,5]. In the primary xylem vessels, *Pectobacterium atrosepticum* (*Pba*), has been shown to form specific multicellular biofilm-like structures known as bacterial emboli, in which bacteria reside in an extracellular matrix [4,6]. In contrast to typical biofilms, the matrix of which consists of bacterial exopolysaccharides (EPS), the initial matrix of bacterial emboli is composed of the plant cell wall polysaccharide fragments (predominantly rhamnogalacturonan I, RG-I). RG-I fragments are released from the plant cell wall into the vessel lumen due to the specific pathogen-induced plant reaction and consolidate individual *Pba* cells in a holistic structure [7,8]. Although the RG-I fragments enable *Pba* cells to initialize the formation of bacterial emboli, RG-I is destroyed as the bacterial embolus is developed. However, despite that, an extracellular polymeric network continues to provide the structural integrity of the “mature” bacterial embolus.

In our previous study, we showed for the first time that *Pba* is able to produce EPS which constitute the bacterial embolus matrix, substituting the RG-I matrix at the advanced stages of their development [9]. *Pba* EPS are the polymers of 100 to ˃400 kDa with a branched structure. Their backbones consist of [→3)-α-D-Gal*p*-(1→2)-α-D-Man*p*-(1→4)-α-L-Rha*p*-(1→] and the side chains, which contain specific 10-carbon branched monosaccharide erwiniose (Erw), are composed of Erw-(1→3)-α-D-Gal*p*-(1→. The side chains are attached to the mannopyranosyl residue of the backbone at the O-3 position, and the galactopyranosyl residues of the side chains are acetylated at O-2 position.

Other members of the SRP, the species of the *Dickeya* genus (formerly *Erwinia chrysanthemi*), were also shown to produce EPS. Herewith, different strains were shown to produce EPS of different monosaccharide compositions: (1) Rha, Glc, Man, and GlcA (3:1:1:1); (2) Rha, Gal, and GalA (4:1:1); and (3) Fuc, Gal, Glc, and GlcA (2:2:1:1) [10,11,12,13,14].

In general, bacterial EPS carry out several functions. EPS constitute a major portion of the extracellular matrix of biofilms [15,16]. For this, EPS form supramolecular networks, in which bacterial cells are retained and effectively implement communicative behavior [17,18]. Bacterial strains, including phytopathogenic strains, that are deficient in EPS production have been widely shown to have reduced biofilm-forming capacity, as well as reduced virulence [19,20,21]. The synthesis of EPS and the formation of biofilms (or biofilm-like structures) are of particular importance for phytopathogenic bacteria that colonize xylem vessels. The intensive xylem sap flow can negatively affect bacterial communication and the synthesis of virulence factors. In turn, blockage or a reduction in water flow by the EPS/biofilm can enable pathogens to effectively colonize vessels and to interact with the host plant.

In addition to their structure-forming capacity, EPS carry out protective functions. These polymers preserve bacteria from desiccation and toxic compounds [22,23,24]. EPS have been also widely shown to cause the detoxification of reactive oxygen species (ROS). Herewith, the enrichment of EPS with various electron-donating functional groups may provide a direct reduction of ROS [25]. EPS can also repress ROS-synthesizing enzymes and chelate the Ca^2+^ that serves as a secondary messenger inducing ROS accumulation and the Fe^2+^ required for the Fenton reaction yielding the hydroxyl radical [26,27]. EPS can also prevent the agglutination of bacteria by the host plant agglutinins during infection [28,29,30].

EPS also play a role in phytoimmunity. Xanthan (the EPS produced by *Xanthomonas* species) can repress the hypersensitive response (HR)-like reactions (strong defense reactions associated with programmed cell death) [31,32]. In contrast, the EPS of some phytopathogenic bacteria can act as elicitors (PAMP, pathogen-associated molecular pattern) themselves, inducing phytoalexin synthesis, ROS accumulation and stomatal closure [33,34,35].

Although EPS are well-known as multifunctional polymers that grant many benefits to bacteria and participate in plant–microbe interactions, almost no information exists about the properties of the EPS of the SRP. The only exception is the finding that the EPS of different *Dickeya* strains provide the increase of the liquid viscosity [36].

Therefore, our study aimed to elucidate the properties of *Pba* EPS from the perspective of their potential role in *Pba*–plant interactions. Herewith, we gave special attention to those features of EPS that are of special importance for bacterial embolus development, namely their structure-forming capacity and their detoxification of ROS, the level of which is increased during the formation of bacterial emboli [7]. Additionally, we assessed the phytoimmune properties (both inducing and suppressive) of the target polymers.

## 2. Results

### 2.1. The Viscosity of Pba EPS Solutions

**Figure 1:**
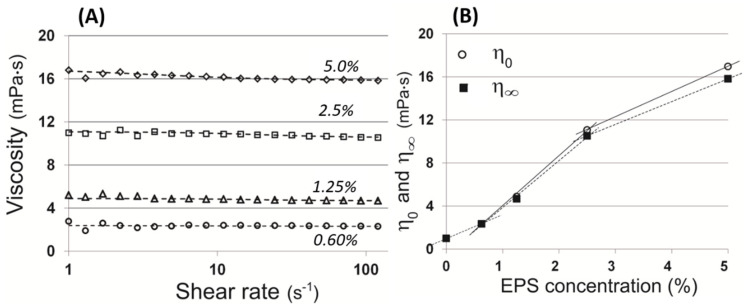
The viscosities of *Pectobacterium atrosepticum* exopolysaccharides (*Pba* EPS) solutions. (**A**) The dependence of the apparent viscosity on the shear rate fitted by the Cross-equation. (**B**) The dependence of zero (η_0_) and infinite (η_∞_) shear rate viscosities on the concentration of *Pba* EPS. The measurements were performed at 20 °C. The viscosity of distilled water (1.002 mPa∙s) was used to designate the viscosity at a null *Pba* EPS concentration.

At higher *Pba* EPS concentrations (1.25–5.0%), the solutions exhibited a shear thinning behavior (non-Newtonian pseudoplastic fluid): the increase in the shear rate led to a decrease the viscosity (Figure 1A). However, at a lower *Pba* EPS concentration (0.60%) the solution displayed Newtonian behavior (the increase in the shear rate did not lead to a decrease in the viscosity). To determine the highest viscosity rates of the analyzed solutions, the viscosity at a zero shear rate (η_0_) was calculated by fitting of a Cross-equation (Table 1, Figure 1A) [37].

The analysis of the dependence of the zero and infinite shear rate viscosities on the *Pba* EPS concentration showed that the non-Newtonian behavior of the *Pba* EPS solutions manifested as the concentration of *Pba* EPS increased (Figure 1B). Herewith, the difference between the zero and infinite shear rate viscosities was small (less than 7% for the 5% *Pba* EPS concentration).

### 2.2. Formation of Supramolecular Aggregates of Pba EPS

**Figure 2:**
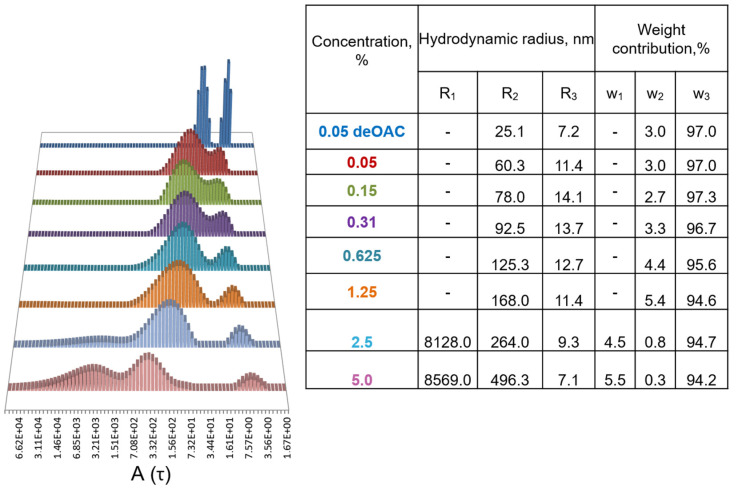
The hydrodynamic radius and weight contribution of particles of *Pectobacterium atrosepticum* exopolysaccharides (*Pba* EPS) at different concentrations of the polymers. The distribution of the decay times as a function of concentration, A(τ). The weight contribution of each particle type to the total light scattering was calculated using the Shibayama’s theory [38]. The distribution functions of the decay time were obtained by the distribution analysis multi-pass algorithm for all concentrations of *Pba* EPS at scattering angle of 150°. deOAc 0.05 shows the results for 0.05% deacetylated *Pba* EPS.

At a low *Pba* EPS concentration (0.05%), the polymer formed two types of particles with mean hydrodynamic radii of 11.4 (small particles, R3) and 60.3 nm (medium particles, R2) (Figure 2). The hydrodynamic radius of the medium particles increased monotonically as the concentration of *Pba* EPS rose in the solution (60.3, 78.0, 92.5, 125.3, 168.0, 264.0, and 496.3 nm at concentration of 0.05, 0.15, 0.31, 0.62, 1.25, 2.5, and 5.0%, respectively). In addition, at higher concentrations (2.5 and 5.0%), large particles of ~8000 nm (R1) formed in the *Pba* EPS solutions (Figure 2).

The weight contribution of different particle types to the total light scattering varied depending on the *Pba* EPS concentration (Figure 2). The weight contribution of small particles (~10 nm) decreased following an increase in the *Pba* EPS concentration, while the weight contribution of the medium particles (78.0–496.3 nm) increased up to a concentration of 1.25%. At higher concentrations, when large particles of ~8000 nm emerged, the weight contribution of the medium particles decreased. This means that large particles formed due to the aggregation of medium particles (Figure 2).

To obtain information about the “elementary particles” of *Pba* EPS, we tried to break the *Pba* EPS aggregates by heating (90 °C), high osmolarity (3 M KCl), and sonication (37 kHz, 80 °C, 1 h). However, these treatments did not influence the hydrodynamic radius of *Pba* EPS aggregates. Given that the Gal residues of the side chains of *Pba* EPS are substituted by acetyl groups, we presumed that these groups might assist in the formation of the aggregates of the target polymers. To check this hypothesis, a deacetylated form of *Pba* EPS was obtained and analyzed by dynamic light scattering. The deacetylated *Pba* EPS also formed two types of particles that, however, had lower hydrodynamic radii (7.2 and 25.1 nm) than that of the native (acetylated) polymer (11.4 and 60.3 nm) (Figure 2). This means that acetyl groups contribute significantly to the formation of *Pba* EPS aggregates.

### 2.3. Antioxidant Properties of Pba EPS

**Figure 3:**
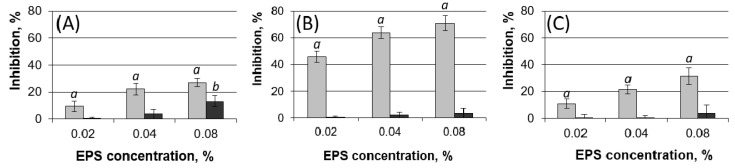
The ROS scavenging activity of native (light gray) and deacetylated (dark gray) exopolysaccharides of *Pectobacterium atrosepticum* (*Pba* EPS). The scavenging of hydroxyl radicals (**A**) and superoxide radicals (**B**) were analyzed as well as the influence of *Pba* EPS on the lipid peroxidation (**C**). ROS scavenging activity was expressed as the level of inhibition of substrate oxidation (salicylic acid (**A**), pyrogallol (**B**), lipids (**C**)) in ROS-generating systems in vitro. Water instead of *Pba* EPS was added to the control variant (0% inhibition of substrate oxidation). The experiments were performed in five replicates. Lowercase letters “*a*” or “*b*” show significant differences from the control (Mann–Whitney two-sided test, *p* < 0.05), where values marked with“*a*” and “*b*” show a significant difference from each other.

*Pba* EPS repressed the oxidation of salicylic acid by hydroxyl radicals by 10, 22, and 27% at concentrations of 0.02, 0.04, and 0.08% *Pba* EPS, respectively (Figure 3A). The deacetylated *Pba* EPS showed lower repression of salicylic acid oxidation only at the highest concentration applied. The most pronounced scavenging activity of *Pba* EPS was observed towards the superoxide radical. The autoxidation of pyrogallol was repressed by *Pba* EPS by 46, 64, and 71% at concentrations of 0.02, 0.04, and 0.08% *Pba* EPS, respectively (Figure 3B). The deacetylated *Pba* EPS did not repress the autoxidation of pyrogallol. *Pba* EPS (but not its deacetylated form) also decreased the lipid peroxidation level by 11, 22, and 32% at concentrations of 0.02, 0.04, and 0.08% *Pba* EPS, respectively (Figure 3C).

**Figure 4:**
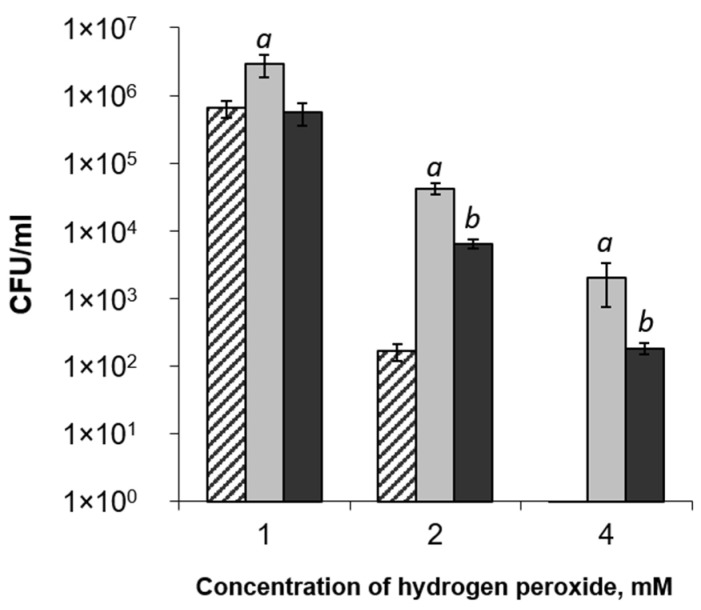
The influence of exopolysaccharides of *Pectobacterium atrosepticum* (*Pba* EPS) on *Pba* cell tolerance to hydrogen peroxide. Different aliquots of *Pba* cells (~10^8^ CFU/mL) in a carbon-deficient medium were supplemented with water (control, hatched columns) or 0.05% *Pba* EPS (light gray columns) or deacetylated *Pba* EPS (dark gray columns). Each variant was supplemented with water or hydrogen peroxide (1, 2, or 4 mM). The cell titer was determined 24 h after treatment. The experiments were performed in three biological replicates. Lowercase letters “*a*” or “*b*” show a significant difference from the control (Mann–Whitney two-sided test, *p* < 0.05), while values marked with “*a*” and “*b*” show a significant difference from each other.

Exogenously added *Pba* EPS (0.05%) increased the tolerance of *Pba* cells to oxidative stress (hydrogen peroxide). In the presence of 4 mM hydrogen peroxide, no CFUs were revealed in the suspensions of cells that were not treated with *Pba* EPS; the CFU titer in the *Pba* EPS-treated cell suspensions was 2 × 10^3^ CFU/mL (Figure 4). At lower hydrogen peroxide concentrations (1 and 2 mM), the CFU titer in the *Pba* EPS-treated suspensions was 5 and 250 times higher, respectively, than that in the cell suspensions not treated with *Pba* EPS. The deacetylated *Pba* EPS also protected *Pba* cells from hydrogen peroxide; however, the CFU titer in the *Pba* EPS-treated suspensions was 7 and 10 times higher, respectively, than in cell suspensions treated with deacetylated *Pba* EPS (Figure 4).

Taken together our results show that *Pba* EPS have pronounced antioxidant properties that are determined (at least partly) by the acetyl groups present in the polymers’ composition.

### 2.4. Phytoimmune Properties of Pba EPS

**Figure 5:**
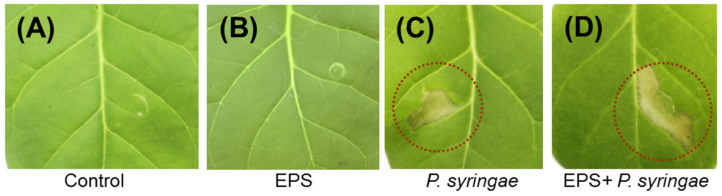
The role of *Pectobacterium atrosepticum* (*Pba* EPS) in the hypersensitive response (HR) induced by *Pseudomonas syringae* in tobacco plants. Leaves were infiltrated with 10 mM MgSO_4_ (control, **A**), 0.05% *Pba* EPS (**B**), ~10^8^ CFU/mL *P. syringae* in 10 mM MgSO_4_ (**C**), and both *Pba* EPS and *P. syringae* (**D**). In the latter treatment (**D**), *Pba* EPS was infiltrated 12 h before the infiltration of *P. syringae*. The experiment was performed in three biological replicates. Photos were taken 3 days after treatment. Dotted circles indicate the area where the HR was manifested. Some differences in the area of the programmed cell death in (**C**,**D**) were not related to the enhanced/decreased HR but were associated with differences in the efficiency of the infiltration of solutions in each particular case.

The infiltration of *Pba* EPS into tobacco leaves was not associated with any visual manifestation (except for slight mechanical damage at the syringe application site), including signs of HR (Figure 5A,B). In turn, the infiltration of *Pseudomonas syringae* cells caused pronounced HR (Figure 5C). *Pba* EPS did not repress the HR caused by *P. syringae* when the polymers were infiltrated into leaves 12 h before treatment with *P. syringae* (Figure 5D). This means that *Pba* EPS neither induced nor repressed the qualitative resistance related to the manifestation of the HR.

**Figure 6:**
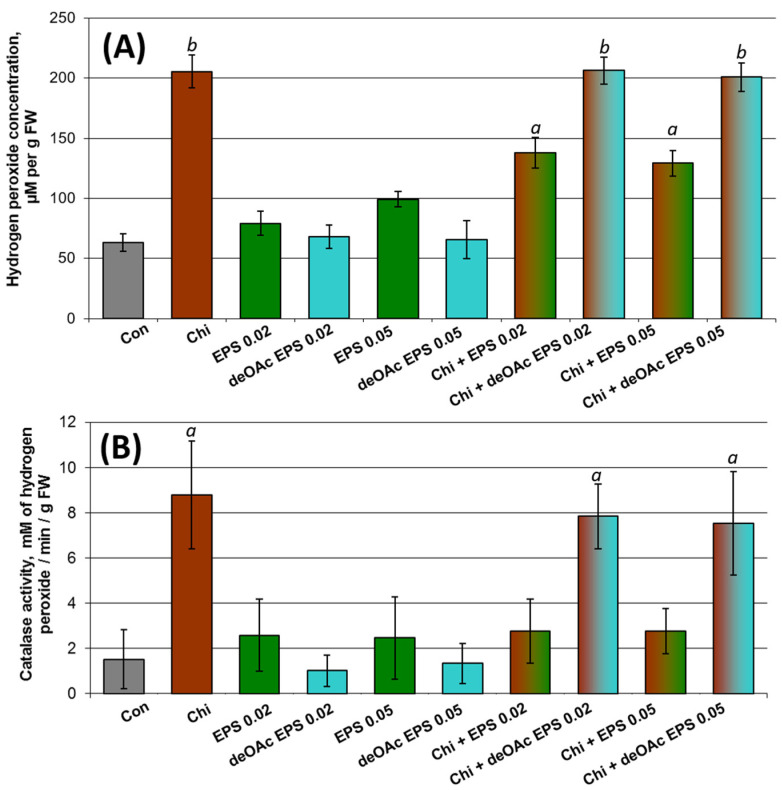
The role of the exopolysaccharides of *Pectobacterium atrosepticum* (*Pba* EPS) in PAMP-triggered immunity. The levels of hydrogen peroxide (**A**) and catalase activity (**B**) in tobacco leaves were measured after infiltration with water (Con, gray), 1 µM chitohexaose (Chi, brown), 0.02% or 0.05% *Pba* EPS (EPS 0.02/EPS 0.05, green), 0.02% or 0.05% deacetylated *Pba* EPS (deOAc EPS 0.02/deOAc EPS 0.05, blue), both 1 µM chitohexaose and 0.02% or 0.05% *Pba* EPS (Chi + EPS 0.02/Chi + EPS 0.05, brown–green), both 1 µM chitohexaose and 0.02% or 0.05% deacetylated *Pba* EPS (Chi + deOAc EPS 0.02/Chi + deOAc EPS 0.05, brown–blue). In the latter two treatments, EPS/deOAc EPS were infiltrated 12 h before the treatment with chitohexaose. Hydrogen peroxide and catalase activity were measured 6 h after treatment in five biological replicates. Lowercase letters “*a*” or “*b*” show a significant difference from the control (Mann–Whitney two-sided test, *p* < 0.05), while values marked with “*a*” and “*b*” show a significant difference from each other. FW—fresh weight.

Since quantitative resistance is associated with an increase in hydrogen peroxide levels and the induction of antioxidant systems (including catalase activity), these parameters were used to investigate whether *Pba* EPS takes part in PAMP-triggered immunity, which is induced by different elicitors (PAMP), including chitooligosaccharides. The infiltration of *Pba* EPS into tobacco leaves did not lead to a significant increase in hydrogen peroxide levels or the induction of catalase activity (Figure 6). The deacetylated *Pba* EPS also did not influence the analyzed parameters. Chitooligosaccharides (chitohexaose) infiltration resulted in both the accumulation of hydrogen peroxide and the induction of catalase activity (Figure 6). Herewith, when *Pba* EPS-pretreated leaves were infiltrated with chitohexaose, the increase in hydrogen peroxide levels was much lower than that in leaves not pretreated with *Pba* EPS after chitohexaose infiltration. Catalase activity was not induced at all when infiltration with chitohexaose was followed by the *Pba* EPS pretreatment. In turn, when the leaves were pretreated with the deacetylated *Pba* EPS, the chitohexaose infiltration led to the accumulation of hydrogen peroxide and induction of the catalase activity to the same degree as in leaves not pretreated with *Pba* EPS (Figure 6). Thus, *Pba* EPS repressed the quantitative resistance induced by PAMP (chitohexaose); this repressive property depends (at least partly) on the presence of the acetyl groups typical of native *Pba* EPS.

## 3. Discussion

In the present study, we investigated whether *Pba* EPS has the potential to facilitate the interaction of *Pba* with a host plant. We gave special attention to the properties that are of special importance for the development of bacterial emboli: the “multicellular” structures that are formed by *Pba* cells in the primary xylem vessels [4,6]. First, given that the polymer (the RG-I of the host plant) that constitutes the initial matrix of bacterial emboli is destroyed as the bacterial emboli “mature”, the *Pba* EPS, the polymers that substitute RG-I within the bacterial embolus matrix, should possess structure-forming capacity and form supramolecular networks to maintain the structural integrity of the bacterial emboli. Second, since bacterial embolus development is associated with an increased level of ROS in the primary vessels [7], the presence of metabolites with antioxidant properties within the bacterial embolus matrix is of particular importance for *Pba* cells.

To analyze whether intermolecular interactions are typical of *Pba* EPS, the viscosity of *Pba* EPS solutions and the ability of the polymers to form supramolecular aggregates were investigated. Our results showed that *Pba* EPS significantly increased the viscosity of the water solution and the *Pba* EPS solutions with concentrations of more than 1.25% displayed a shear thinning behavior. We compared the rheological properties of the solutions of *Pba* EPS (this study) and the EPS of other phytopathogenic bacteria (previously published data) (Table 2). The highest levels of viscosity (zero shear rate viscosity, η_0_) was provided by the xanthan produced by *Xanthomonas* species and EPS from *Pantoea* sp., and were greater than the zero shear rate viscosity of the *Pba* EPS solution by three or four orders of magnitude. Herewith, the solutions of alginate from *Pseudomonas oleovorans* are comparable with the solutions of *Pba* EPS in terms of their viscous properties. Interestingly, the shear thinning behavior of the solutions of different EPS manifested to different degrees. For example, a 0.4% solution of xanthan has a zero shear rate viscosity of 39,000 mPa·s but its infinite shear rate viscosity is more than 600 times lower (62 mPa·s) [39]. A similar pronounced tendency was also noted for solutions of levan from *Erwinia amylovora* and EPS from *Pantoea* sp. [40,41] (Table 2). For *Pba* EPS, the difference between the zero and infinite shear rate viscosity was less than 7%. A similar situation was observed for alginate from *P. oleovorans* [42]. The small difference between the zero and the infinite shear rate viscosity “equalized” *Pba* EPS with some other EPS in terms of the viscous properties of their solutions. For example, the zero shear rate viscosity of 6% levan from *Brenneria* sp. (643 mPa·s) was almost 38 times greater than the viscosity of 5% *Pba* EPS; however, here, the infinite shear rate viscosity of *Pba* EPS was even greater (16 mPa·s) than that of levan from *Brenneria* sp. (12 mPa·s). Similarly, the zero and infinite shear rate viscosity of a 2% solution of succinoglycan from *Agrobacterium radiobacter* (112 and 25 mPa·s, respectively) differed by 4.5 times, while the same parameters for the 2.5% solution of *Pba* EPS (11.1 and 10.9, respectively) differed only by 1.5%. In other words, the resting structure of the *Pba* EPS solution (as well as *P. oleovorans* alginate) did not differ significantly from the ordered solution’s structure that emerged due to the share stress that can be imposed, particularly by liquid flow. This means that some EPS (including *Pba* EPS), although they provide rather low viscosity to the solutions (at least compared with some other EPS), can maintain the viscosity irrespective of the intensity of the water flow. This property of EPS seems to enable bacteria to withstand the water flow, which is beneficial for the phytopathogens that colonize the water-conducting xylem vessels of the host plant.

In addition to conferring liquid viscosity, *Pba* EPS are able to form supramolecular aggregates that can also provide the structural integrity of bacterial emboli. The size of the *Pba* EPS aggregates as well as the weight contribution of larger aggregates increase as the concentration of the polymer rises. The formation of aggregates is achieved (at least partly) by the acetyl groups attached to the galactopyranosyl residues of the side chains of the target polymers; the deacetylation of *Pba* EPS reduces their ability to aggregate.

The ability to form aggregates has been also demonstrated for the EPS of phytopathogenic bacteria other than *Pba*: *Rhizobium radiobacter*, *Xanthomonas* sp., and *Brenneria* sp. [44,47,48]. For *Brenneria* sp. EPS, the particle sizes were around 90 nm and no significant differences in the sizes were observed at polymer concentrations of 0.1% and 1%. The EPS of *R. radiobacter* and *Xanthomonas* sp. formed rather large aggregates with hydrodynamic radii of 1000 nm and 800 nm, respectively. However, whether the particle sizes change at different polymer concentrations remains unknown, since the only one concentration has been analyzed for each polymer (0.5% EPS of *R. radiobacter* and 0.2% xanthan of *Xanthomonas* sp.). We considered a range of concentrations of *Pba* EPS (0.05–5%) in terms of the formation of aggregates. This allowed us to describe the dynamics of *Pba* EPS particles at increasing polymer concentrations and to reveal very large molecular aggregates with a hydrodynamic radius of ~8000 nm.

Unfortunately, we could not determine the exact concentration of *Pba* EPS in the infected plant, especially the local concentrations within the matrix of the bacterial emboli or around the *Pba* cells’ surface. However, considering the content of *Pba* EPS in the cultures in vitro, the differences in the cell density in vitro and in planta, the increased synthesis of *Pba* EPS in planta compared with in vitro, and the high bacterial cell density within the bacterial emboli [4,9], the analyzed concentrations (including the largest ones) are likely to be achieved within particular compartments of the pathosystem. Taken together, *Pba* EPS indeed have the structure-forming capacity and form supramolecular networks that can maintain the structural integrity of the bacterial emboli.

*Pba* EPS, in addition to their structure-forming capacity, possess pronounced antioxidant properties. Our data showed that *Pba* EPS repressed lipid peroxidation and served as scavengers for hydroxyl radicals and superoxide radicals. We also demonstrated that treating *Pba* cells with *Pba* EPS reduced the damage caused by hydrogen peroxide. The antioxidant properties of *Pba* EPS revealed here were partly provided by the acetyl groups present in the polymer’s composition; the deacetylation of *Pba* EPS reduced their ROS-scavenging activity. ROS-scavenging activity has been widely shown for the EPS of different bacteria [25,49,50]; however, among the EPS of phytopathogenic bacteria, only the xanthan and EPS of *P. agglomerans* have been shown to possess such properties [51,52].

*Pba* EPS were also shown in our study to possess immune properties. The target polymers did not mediate the HR: *Pba* EPS neither induced nor repressed this type of immunity. In contrast, xanthan produced by *X. campestris* has been shown to suppress HR-like reactions in *Arabidopsis*, *Nicotiana bethamiana*, and rice [32]. *Pba* EPS did not show PAMP properties: after infiltration into tobacco leaves, these polymers did not induce hydrogen peroxide accumulation and catalase activity—the typical hallmarks of PAMP-triggered immunity. The EPS of some phytopathogenic bacteria (*P. syringae*, *X. campestris*, and *Ralstonia solanacearum*) have been shown to display PAMP properties and induce immune responses such as the accumulation of ROS, the synthesis of phytoalexins, and stomatal closure [33,34,53,54]. However, we did not find evidence that *Pba* EPS are recognized by plant immune systems. The treatment of axenically grown tobacco plants with *Pba* EPS (0.02 or 0.05%) before inoculation with *Pba* cells did not reduce disease development, indicating that *Pba* EPS did not act as elicitors.

In contrast, we have shown that *Pba* EPS repressed the immunity triggered by chitooligosaccharides—a well-known PAMP. Herewith, the acetyl groups made a large contribution to the immunosuppressive properties of *Pba* EPS. The suppression of PAMP-triggered immunity has also been demonstrated for EPS synthesized by the phytopathogenic bacteria *X. campestris*, *P. syringae*, *E. amylovora*, and *R.* *solanacearum*; the deacetylated EPS of *X. campestris* had reduced immunosuppressive activity compared with the native EPS [55]. Moreover, mutant strains of various phytopathogenic bacteria that are deficient in the production of EPS have been widely shown to activate host plant defenses more strongly than the corresponding wild-types [22,27,56,57,58,59].

Thus, our study showed that *Pba* EPS possess properties that may contribute to *Pba* in plant colonization and the formation of bacterial emboli. *Pba* EPS provides viscosity to the liquid, although this polymer is far from being a “leader” in these terms compared with the EPS of some other phytopathogenic bacteria. However, this level of viscosity is likely to be enough for the maintenance of bacterial emboli: given that the initiation of the bacterial embolus assemblage is provided by RG-I rather than *Pba* EPS, the requirements regarding the viscous properties of *Pba* EPS might be lower than those of EPS that initiate the formation of bacterial biofilms. The structure-forming properties of *Pba* EPS are enriched by the ability of these polymers to form large aggregates that presumably provide structural integrity for the mature bacterial emboli. *Pba* EPS also display pronounced antioxidant properties that are of particular importance, since the development of bacterial emboli is coupled with ROS accumulation. In addition, *Pba* EPS act as immunosuppressors that repress PAMP-triggered immunity. Thus, *Pba* EPS should be considered as virulence factors of *Pba*. Given that *Pba* EPS emerge within the bacterial embolus matrix before the complete destruction of the host plant-derived RG-I, it would be interesting to assess whether these two polymers are able to form heterocomplexes, and, if so, to analyze the properties of the *Pba* EPS–RG-I heterocomplexes. In addition, to get better insight into the role of *Pba* EPS in plant–microbe interactions, an analysis of EPS-deficient *Pba* mutant is required.

## 4. Materials and Methods

### 4.1. Collection of the Pba EPS Samples

Since the synthesis of EPS by *Pba* in vitro is induced by starvation, EPS samples were obtained from the supernatants of the staving *Pba* cultures according to the previous protocol [9]. Briefly, early stationary phase *Pba* cells were washed twice, resuspended, and then incubated for 14 days in a carbon-free AB medium (1.0 g/L NH_4_Cl; 0.62 g/L MgSO_4_·7H_2_O; 0.15 g/L KCl; 0.013 g/L CaCl_2_·2H_2_O, pH 7.5). A bulk of the cells was removed from the cultures by centrifugation (14,000× *g*, 10 °C, 10 min). The remaining cells were removed by filtration through nitrocellulose filters (0.2 μm; Millipore, Germany). The cell-free supernatants were incubated at 100 °C for 10 min to denature the proteins, and then centrifuged again and filtered through nitrocellulose filters. The resulting supernatants were concentrated 50–100 times using a vacuum evaporator RV 8 V (IKA, Staufen, Germany) at 80–90 °C and then the samples were dialyzed (cellulose membrane, 14 kDa, Sigma-Aldrich, St. Louis, MO, USA) against deionized water. The dialyzed samples were concentrated up to 1 mL volume using an Eppendorf Concentrator Plus (Eppendorf, Germany). The target *Pba* EPS fraction was separated by size-exclusion chromatography on a Sepharose CL-4B column (1.2 × 40 cm, Pharmacia, Uppsala, Sweden) using a 0.01 M pyridine/acetic acid solution (pH 5.0). The carbohydrate content in each fraction was measured using the phenol–sulfuric acid assay [60]. A fraction corresponding to an elution volume of 11–21 mL (100 -> 400 kDa) that contained the target polymers was collected. The monosaccharide content of the target fraction was verified by high-performance anion-exchange chromatography on a CarboPac PA-1 column (4 × 250 mm; Dionex, Sunnyvale, CA, USA), using pulse-amperometric detection (Dionex). Herewith, polysaccharides of the fraction obtained after size-exclusion chromatography were hydrolyzed with 2 M trifluoroacetic acid (TFA; Sigma, St. Louis, MO, USA) at 120 °C for 1 h [61], dried in a stream of air at 60 °C, and redissolved in deionized water before the analysis. To obtain O-deacetylated EPS, samples of native *Pba* EPS were incubated in 12% NH_4_OH at 37 °C for 16 h [9].

### 4.2. Rheological Measurements

The viscosities of the *Pba* EPS solutions (0.62, 1.25, 2.5, and 5.0 %) in deionized water were measured on rheometer a MCR 102 rheometer (Anton Paar, Graz, Austria) equipped with Peltier (H-PTD200) temperature control system; parallel-plate geometry was used with a plate diameter of 50 mm and a gap of 0.295 ± 0.065mm. The viscosity was measured at shear rates of 0.1–120 s^−1^. Calibration with a viscosity standard liquid (Mendeleyev Institute for Metrology, Russia) showed agreement within the analyzed shear rates, with an error of ~0.5%. The experimental data were approximated by the Cross-equation [37], which is written as:

$$\eta =\frac{\eta_{0}-\eta_{\infty }}{1+{\left(\lambda \dot{\gamma }\right)}^{n}}+\eta_{\infty }$$

where η_0_ and η_∞_ are the zero and infinite shear rate viscosities, respectively; λ is a characteristic time of the solution; and n is a rate index.

The parameters of the Cross-equation were calculated using RheoCompass software (Anton Paar, Graz, Austria).

### 4.3. Dynamic Light Scattering

The hydrodynamic radii of native (at the concentrations of 0.05, 0.15, 0.31, 0.62, 1.25, 2.5, and 5%) and O-deacetylated (0.05%) *Pba* EPS were measured by a spectrometer Photocor Complex (Photocor Instruments Inc., Moscow, Russia) equipped with a compact goniometer, a real-time correlator (200 channels; fastest sampling period 10 ns), a thermostat, and a monochromatic laser light (λ) operating at 657.29 nm. All measurements were performed in deionized water (Type A) at 20 °C and a scattering angle of θ = 150°. Autocorrelation functions were recorded during a 40–120 s accumulation time using Photocor software. Each autocorrelation function was averaged from 25–30 measurements. The data were processed by the distribution analysis multi-pass algorithm using DynaLS software. Before the analysis, the solvent and samples were filtered through a 0.22 μm polytetrafluoroethylene (PTFE) membrane. To calculate the particle sizes, the standard values of viscosity and the refractive index of water at 20 °C were used. The z-averaged hydrodynamic radius, R_h_, was calculated from the Stokes–Einstein relation as follows:

$$R_{h}=\frac{k_{B}T}{6\pi \eta D}$$

where η is the viscosity of the solvent, k_B_ is the Boltzmann constant, T is the absolute temperature, and D is the diffusion coefficient. The weight contribution of each particle type to the total light scattering was calculated according to the Shibayama’s theory [38].

### 4.4. Reactive Oxygen Species Scavenging Assays

*Hydroxyl radical scavenging assay.* The hydroxyl radicals were generated in a H_2_O_2_–FeSO_4_ system by oxidation of FeSO_4_ and were assayed by the change in color of salicylic acid. The hydroxyl radicals were generated in 3.0 mL of reaction mixture containing 25 mM FeSO_4_, 2 mM sodium salicylate, 6 mM H_2_O_2_, and the tested solutions: *Pba* EPS (native or deacetylated) at concentrations of 0.02, 0.04, and 0.08% or water (control). The mixtures were incubated at 37 °C for 1 h. The change in absorbance was measured at 510 nm [51].

*Superoxide radical scavenging assay.* Superoxide radicals were generated in a system of pyrogallol autoxidation under alkalescent conditions. The reaction was performed in 3.0 mL of Tris-HCl buffer (50 mM, pH 8.2), which contained 3 mM pyrogallol and the test solutions: *Pba* EPS (native or deacetylated) at concentrations of 0.02, 0.04, and 0.08% or water (control). The change in absorbance was measured at 325 nm [51].

*Lipid peroxidation assay.* The yolk taken from an egg was added to an equal volume of pH 7.45 PBS and stirred vigorously on a magnetic stirrer, then diluted with a 40× volume of PBS to prepare a yolk suspension. Next, 0.5 mL of the yolk suspension was incubated at 37 °C for 15 min with the test solutions (*Pba* EPS (native or deacetylated) at concentrations of 0.02, 0.04, and 0.08% or water) and 6 mM FeSO_4_ in 2-mL of PBS. The reaction was stopped by 0.5 mL of 20% trichloroacetic acid and then the sample was heated at 100 °C for 15 min with 1 mL 0.8% 2-thiobarbituric acid. The reaction products were measured at 532 nm [51].

The optical densities were measured using a PB2201B spectrophotometer (SOLAR, Belarus). The inhibition of ROS-mediated oxidation of the substrates by EPS was calculated as follows: Inhibition rate (%) = (A−B)/A × 100%, where A is the absorbance of the control groups in the ROS generation systems and B is the absorbance of the test groups. The presented data are the means ± SD of five replicates.

To assess whether *Pba* EPS protected bacterial cells from hydrogen peroxide, early stationary phase *Pba* cells were washed twice and resuspended in a carbon-deficient AB medium up to a density of ~10^8^ CFU/mL and aliquoted. Different aliquots were supplemented with 1/4 volume of water or 2% *Pba* EPS, or 2% deacetylated *Pba* EPS, giving a final concentration of 0.5% of *Pba* EPS or deacetylated *Pba* EPS. Each variant was supplemented with water or hydrogen peroxide (1, 2, or 4 mM). The suspensions were incubated at 28 °C for 24 h; after that, the CFU titer was determined. The experiments were performed in three biological replicates.

### 4.5. Analysis of the Phytoimmune Properties

The phytoimmune properties of *Pba* EPS were analyzed using tobacco plants (*Nicotiana tabacum* Petit Havana SR1). Plants were grown in soil (Peter Peat, Dzerzhinsky, Russia) in 50 mL pots during 4 weeks before the analysis.

To assess the ability of *Pba* EPS to induce or repress the hypersensitive response (qualitative resistance), plant leaves were infiltrated (in three biological replicates) with 100 µL of (A) sterile MgSO_4_ (control), (B) 0.05% *Pba* EPS, (C) *Pseudomonas syringae* DSM 50256 cells suspended in MgSO_4_ up to a density of ~10^8^ colony forming units per milliliter (CFU/mL), (D) both 0.05% *Pba* EPS and *P. syringae* cells. In the latter treatment (D), the EPS was infiltrated 12 h before the infiltration of *P. syringae* cells; herewith, the corresponding control variants were pretreated with sterile MgSO_4_ 12 h prior to infiltration with *P. syringae*. The formation of the hypersensitive response was assessed visually 1–3 days after treatment.

To assess the ability of *Pba* EPS to induce or repress quantitative resistance (PAMP-triggered immunity), plant leaves were infiltrated (in five biological replicates) with (1) sterile water (control), (2) 0.02 or 0.05% *Pba* EPS, (3) 0.02 or 0.05% deacetylated *Pba* EPS, (4) 1 μM chitohexaose (Carbosynth China Ltd., Suzhou, China), (5) both 1 μM chitohexaose and *Pba* EPS (0.02 or 0.05%); (6) both 1 μM chitohexaose and deacetylated *Pba* EPS (0.02 or 0.05%). In the latter two treatments (5 and 6), the EPS (native or deacetylated) was infiltrated 12 h before the infiltration of chitohexaose; herewith, the corresponding control variants were pretreated with water 12 h prior to infiltration with chitohexaose. Six hours after the treatments, the levels of H_2_O_2_ and catalase activity were measured in the infiltrated parts of the leaves.

H_2_O_2_ levels were determined by a method based on the peroxide-mediated oxidation of Fe^2+^ followed by the reaction of Fe^3+^ with xylenol orange (Sigma, St. Louis, MO, USA) [62]. Leaves (100 mg) were ground in 1 mL of a cold 50 mM borate buffer (pH 8.4) in mortars. The homogenates were centrifuged (7000× *g*, 10 min) and 100 μL of the supernatants was added to 500 μL of the assay reagent (500 mM ammonium ferrous sulfate, 50 mM H_2_SO_4_, 200 mM xylenol orange, and 200 mM sorbitol). The absorbance of the Fe^3+^–xylenol orange complex (*A*560) was detected after 45 min using a PB2201B spectrophotometer (SOLAR, Minsk, Belarus). Standard curves of H_2_O_2_ were obtained for each independent experiment by adding various amounts of H_2_O_2_ to 100 mL of a borate buffer mixed to 500 mL of the assay reagent. Data were normalized and expressed as µmol H_2_O_2_ per gram of fresh weight. The presented data are the means ± SD of five biological replicates.

To determine the levels of catalase activity, leaves (100 mg) were ground in 1 mL of a cold K-phosphate buffer (50 mM, pH 7.0) in mortars. The homogenates were centrifuged (7000× *g*, 10 min) and 10 μL of the supernatants was added to 490 μL of the reaction mixture containing a 50 mM K-phosphate buffer (pH 7.0) and 2 mM H_2_O_2_. The absorbance was measured at 240 nm using a PB2201B spectrophotometer (SOLAR, Minsk, Belarus). Data were normalized and expressed as millimoles of H_2_O_2_ per minute per gram of fresh weight (ε = 43.6 M^−1^cm^−1^). The presented data are the means ± SD of five biological replicates.

## Figures and Tables

**Table 1:** Cross-equation parameters for the solutions with different concentrations of *Pectobacterium atrosepticum* exopolysaccharides (*Pba* EPS). η_0_ and η_∞_ are the zero and infinite shear rate viscosities, respectively; λ is the characteristic time of the solution; n is the rate index.

*Pba* EPS Concentration (%)	η_0_ [mPa∙s]	η_∞_ [mPa∙s]	λ [s]	n [−]
5.0	16.973	15.839	0.2785	0.9557
2.5	11.093	10.546	0.0499	1.3592
1.25	4.893	4.688	0.0566	1.6465
0.625	2.362	2.362	-	-

**Table 2:** The viscosities of the solutions of exopolysaccharides (EPS) of different phytopathogenic bacteria. Columns 2–5 show the viscosity values for the approximate EPS concentrations (0.4–0.6%, 1–1.2%, 2–3%, and 4–10%, respectively); the exact concentration is given for each particular case (presented in brackets in italics). The three viscosity values (mPa·s) given in each cell correspond to the zero shear rate viscosity (η_0_), the viscosity at a shear rate of $\dot{\gamma }=10$ and the viscosity at $\dot{\gamma }=100$ In cases where η_0_ was not presented in published data, the maximum value of the viscosity curve was considered as η_0_. * Erwinia chrysanthemi is now attributed to the *Dickeya* genus.

EPS Name, Bacterial Species	Viscosity at Different Concenrtations (C, %) at Different Shear Rates η_0_$/\eta_{\dot{\gamma }=10}$$/\eta_{\dot{\gamma }=100}(\mathrm{mPa}\cdot s)$				Reference
	0.4 ≤ C ≤ 0.6	1 ≤ C ≤ 1.25	2 ≤ C ≤ 3	4 ≤ C ≤ 10	
EPS, *Pectobacterium atrosepticum*	2.4/2.4/2.4 *(0.6%)*	4.9/4.7/4.8 *(1.25%)*	11/11/11 *(2.5%)*	17/16/16 *(5%)*	This study
EPS80, Erwinia chrysanthemi *	32/32/23 *(0.5%)*				[36]
EPS9, Erwinia chrysanthemi *	112/109/47 *(0.5%)*				[36]
Levan, *Erwinia amylovora*			44/38/33 *(2%)*	101,700/20,600/4387 *(8%)*	[41]
CAS EPS, *Rhizobium radiobacter*			102/102/27 *(2%)*	186/186/43 *(6%)*	[43]
Alginate, *Pseudomonas oleovorans*			-	26/25/25 *(10%)*	[42]
Levan, *Brenneria* sp.			0.6/0.6/0.6 *(3%)*	643/26/12 *(6%)*	[44]
Succinoglycan, *Agrobacterium radiobacter*	29/17/5 *(0.5%)*	37/33/7.4 *(1%)*	112/95/25 *(2%)*		[45]
EPS, *Pantoea* sp.	1250/1250/1250 *(0.5%)*	34,300/17,180/2500 *(1%)*	61,200/30,000/2600 *(2%)*		[40]
Xanthan gum, *Xanthamonas* sp.	39,000/343/62 *(0.4%)*				[39]
EPS S10, *Rhizobium radiobacter*	4400/173/51 *(0.5%)*	1.4 × 10^6^/6373/576 *(1%)*			[46]

## Data Availability

Not applicable.
